# Combined systemic immune-inflammatory index and prognostic nutritional index predict outcomes in advanced non-small cell lung cancer patients receiving platinum-doublet chemotherapy

**DOI:** 10.3389/fonc.2023.996312

**Published:** 2023-04-03

**Authors:** Ruiyun Fan, Ying Chen, Guopeng Xu, Wen Pan, Yantian Lv, Zhongwei Zhang

**Affiliations:** Department of Pulmonary and Critical Care, The Affiliated Suzhou Hospital of Nanjing Medical University, Suzhou Municipal Hospital, Gusu School, Nanjing Medical University, Suzhou, Jiangsu, China

**Keywords:** non-small cell lung cancer, systemic immune-inflammation index, prognostic nutritional index, first-line chemotherapy, survival

## Abstract

**Background:**

Systemic immune-inflammatory index (SII) and prognostic nutritional index (PNI) could evaluate the therapeutic efficacy and prognosis in different tumors. However, no studies investigated the SII-PNI score to predict outcomes in non-small cell lung cancer (NSCLC) patients treated with platinum-doublet chemotherapy. The aim of this study was to investigate the SII-PNI score in predicting outcomes in non-small cell lung cancer (NSCLC) patients treated with platinum-doublet chemotherapy.

**Materials and methods:**

Our study retrospectively analyzed clinical data from 124 patients with advanced NSCLC receiving platinum-doublet chemotherapy. The SII and PNI were calculated based on peripheral blood cell counts and serum albumin, and the optimal cut-off values were determined using receiver operating characteristic (ROC). All patients were divided into three groups according to the SII-PNI score. The association between the SII-PNI score and the clinicopathological characteristics of the patients was examined. The Kaplan-Meier and Cox regression models were used to assess progression-free survival (PFS)and overall survival (OS).

**Results:**

There was no significant correlation between SII, PNI at baseline and chemotherapy response in patients with advanced NSCLC (p>0.05). However, after receiving 4 cycles of platinum-doublet chemotherapy, the SII of the SD group (p=0.0369) and PD group (p=0.0286) was significantly higher than that of the PR group. At the same time, the PNI of the SD group (p=0.0112) and the PD group (p=0.0007) was significantly lower than that of the PR group. The PFS of patients with SII-PNI scores of 0, 1, and 2 were 12.0, 7.0, and 5.0 months, and the OS of patients with SII-PNI scores of 0, 1, and 2 were 34.0, 17.0, and 10.5 months, respectively. There was statistical significance among the three groups (all p <0.001). Multivariate analyses showed that the chemotherapy response of progressive disease (PD) (HR, 3.508; 95% CI, 1.546-7.960; p=0.003) and SII-PNI score of 2 (HR, 4.732; 95% CI, 2.561-8.743; p < 0.001) were independently associated with a shorter OS. The uses of targeted drugs (HR, 0.543; 95% CI, 0.329-0.898; p=0.017) and immune checkpoint inhibitors (HR, 0.218; 95% CI, 0.081-0.584; p=0.002) were protective factors for OS in patients with NSCLC.

**Conclusion:**

Compared with baseline parameters, the correlation between SII, PNI after 4 cycles of chemotherapy and the chemotherapy effect was more significant. The SII-PNI score after 4 cycles of chemotherapy is an effective prognostic biomarker for advanced NSCLC patients treated with platinum-doublet chemotherapy. Patients with a higher SII-PNI score had a worse prognosis.

## Introduction

*Global Cancer Statistics 2020*, released by the International Agency for Research on Cancer (IARC) of the World Health Organization, says that Lung cancer has the second-highest incidence of all malignancies in the world, and it is cancer that causes the highest number of deaths worldwide (1). In China, the morbidity and mortality of lung cancer rank first among all malignant tumors (2). *Global Cancer Statistics 2020* shows that among lung cancer patients diagnosed between 2010 and 2014, 5-year net survival rate in most countries is only 10-20% (1). Non-small cell lung cancer (NSCLC) is the primary histological subtype of lung cancer, accounting for about 80% of all cases. There is no apparent clinical manifestation in the early stage, and about 75% of the patients are in the middle and advanced stages. The choice of NSCLC regimen usually depends on the patient’s clinical stage, pathological type, targeted gene mutations and other factors, and the difference in treatment regimen often leads to different prognosis. But some studies in recent years have supported evidence that patient outcomes are determined not only by tumor characteristics, but also by the patient’s own factors (3–6). Among the factors including physical status, cachexia, inflammatory state and nutritional status, the inflammation, immunity and nutritional index of patients have attracted attention as as prognostic factors and biomarkers to predict response to anti-cancer drugs. Looking for readily available, reliable, and non-invasive prognostic indicators is significant in formulating clinical treatment strategies for patients.

Inflammatory response promotes tumorigenesis and participates in all stages of the tumor. Tumor cells interact with the surrounding stromal and inflammatory cells form an inflammatory tumor microenvironment that drives tumor occurrence, growth, and metastasis (7). Previous studies have proved that there is a correlation between clinical characteristics and survival outcomes of patients with tumors and systemic inflammatory parameters, including neutrophil-to-lymphocyte ratio (NLR), platelet-to-lymphocyte ratio (PLR) (8–10). Systemic immune inflammation index (SII) has been proven to be of great significance in the prognosis of patients with malignant tumors such as hepatocellular carcinoma, breast cancer, renal cell carcinoma, and lung cancer (11–14). Nutritional status is also an essential part of the immune status of tumor patients, which plays a vital role in the survival of tumor patients. Alwarawrah Y et al. have shown that nutritional status during treatment is a crucial factor affecting the chemotherapy effect (15). As a simple and feasible nutritional index, the prognostic nutrition index (PNI) was initially used to evaluate patients’ nutritional and immune status while undergoing gastrointestinal surgery (16), and was later widely used to predict the prognosis of various malignancies, including lung cancer (17–20). SII and PNI are two economical, readily available, and widely used hematological indexes. And the SII-PNI score, as a derivative index of them, can comprehensively reflect the immune status of patients and has been used to evaluate the prognosis of patients with gastric cancer (21–24). However, their clinical significance in patients with advanced NSCLC is not clear. Platinum-based first-line chemotherapy is part of the standard treatment for patients with advanced NSCLC. Although immune checkpoint inhibitor therapy and targeted therapy have revolutionized the treatment of advanced NSCLC, a significant proportion of patients still choose platinum-based chemotherapy due to economic factors, lack of driving gene mutations, and other reasons. The study developed a predictive model that the SII-PNI score predicts the survival outcome of patients with advanced NSCLC receiving platinum-doublet chemotherapy.

## Materials and methods

### Patients

124 patients with NSCLC were collected from April 2015 to April 2022 in the Affiliated Suzhou Hospital of Nanjing Medical University. Inclusion criteria: histologically or cytologically confirmed stage III - IV NSCLC; the initial chemotherapy regimens were cisplatin or carboplatin+pemetrexed or paclitaxel or gemcitabine or docetaxel; receive at least 4 cycles of chemotherapy; the Eastern Cooperative Oncology Group performance status (ECOG PS) was 0-1; treatment options; complete clinical, pathological and laboratory data were available; the follow-up results were complete. Exclusion criteria: patients who had undergone surgical resection before chemotherapy; patients with other systemic malignant tumors; patients with grade II myelosuppression according to the Common Terminology Criteria for Adverse Events (CTCAE) version 5.0; with hematologic diseases and immunodeficiency. The study was approved by the Medical Ethics Committee of Suzhou Municipal Hospital (K-2021-084-H01) and has been confirmed for waiver of informed consent.

### Data collection and definition

Retrospective analysis of the patient’s clinicopathological features from the electronic medical record system. The clinical data include gender, age, smoking history, body mass index (BMI), pathological type, TNM stage, ECOG PS, peripheral blood count and serum albumin before and after four cycles of chemotherapy. According to the eighth edition of the American Joint Committee on Cancer (AJCC) TNM staging system, patients diagnosed with lung cancer were staged. At baseline, hematological indexes were detected within one week before the platinum-doublet chemotherapy and within one week before the fifth cycle after four cycles of chemotherapy. SII = platelet count (×10^9^/L) × neutrophil count/lymphocyte count (×10^9^/L); PNI = serum albumin value (g/L) + 5 × peripheral blood lymphocyte count (×10^9^/L); SII-PNI score: 2, high SII and low PNI; 1: high SII or low PNI; 0, low SII and high PNI.

### Follow up

Patients were followed up through a combination of electronic medical record access systems and telephones, with a follow-up deadline of April 30, 2022. All patients undergo imaging evaluation every 6 to 8 weeks. The progression-free survival (PFS) and overall survival (OS) were calculated according to the follow-up results. After treatment, the patients were followed up regularly, and the curative effect was evaluated according to solid tumor efficacy evaluation (RECIST 1.1). Tumor response can be divided into complete response (CR), partial response (PR), stable disease (SD), and progressive disease (PD). The objective response rate (ORR) was defined as the percentage of the best overall remission confirmed by the investigator, which is the proportion of PR+CR to enrolled patients. The disease control rate (DCR) was defined as the proportion of patients with CR or PR or SD. Progression-free survival (PFS) is defined as the time from the start of chemotherapy to the date of PD, death of any cause. The overall survival time (OS) was calculated as the time from the start of chemotherapy to death or the last follow‐up visit (months).

### Statistical analysis

SPSS 26.0 and GraphPad prism 9.3 statistical software were used for analysis and drawing. The proportion of missing non-laboratory, non-imaging covariates was less than 5%, and the missing observations were deleted from the analysis. Continuous variables are expressed by median (interquartile range) (IQR). The relationship between continuous variables was analyzed by Spearman correlation analysis. The ANOVA test was used to compare differences between three groups. The optimal cut-off value was determined by ROC curve analysis. The Pearson chi-square test was performed to assess the potential correlations between SII-PNI and clinicopathological characteristics. Survival was analyzed using Kaplan–Meier curves, and prognostic differences were examined using the log-rank test. Prognostic factors were assessed using univariate and multivariate analyses (Cox proportional hazard regression model). All *p* values less than 0.05 were considered statistically significant.

## Results

### Patient characteristics

The demographic information and tumor characteristics of 124 patients with advanced NSCLC included in the study are summarized in **Table 1**. There were 95 males (76.6%) and 29 females (23.4%). The median age of the patients was 64 years (IQR:57-69), of which 63 (50.8%) were ≥ 64 years old. There were 53 cases (42.7%) with a history of smoking. There were 8 patients with BMI < 18.5kg/m^2^ (6.5%), 87 patients with 18.5 to 24.9 kg/m^2^ (70.2%), and 29 patients with BMI > 24.9 kg/m^2^ (23.4%). The pathological types were adenocarcinoma in 84 cases (57.7%), squamous cell carcinoma in 37 cases (29.8%), and other types in 3 cases (2.4%), of which stage IV was the most common (75.8%). The ECGO PS of the whole group of patients was 0-1, of which 37 (29.8%) had PS 0 and 87 (70.2%) had PS 1. After at least four cycles of platinum-based chemotherapy, 36 patients had received targeted therapy, 17 patients had received immunotherapy, and 35 patients had received antiangiogenic therapy. After four cycles of chemotherapy, no case reached CR, PR in 25 cases (20.2%), SD in 75 cases (60.5%), and PD in 24 cases (19.4%). The ORR was 20.2%, and the DCR was 80.6%. The median PFS and OS of the patients were 8.0 and 17.0 months, respectively. The SII at baseline is 809.2 (IQR:513.5-1064.2), and the PNI at baseline is 47.7 (IQR:44.2-52.9). After four cycles of chemotherapy, SII was 405.2 (IQR:287.6-718.9), and PNI was 43.9 (IQR:40.5-52.3). At the same time, both at baseline (r=-0.203, p =0.024) and after four cycles (r=-0.250, p=0.005) of chemotherapy, SII and PNI were negatively correlated.

**Table 1:** Clinical characteristics of the patients.

Characteristics	No. (%)
Gender	
Male	95 (76.6)
Female	29 (23.4)
Age (year)	
<64	61 (49.2)
≥64	63 (50.8)
Smoking history	
Ever	53 (42.7)
Never	71 (57.3)
BMI	
<18.5	8 (6.5)
18.5–24.9	87 (70.2)
>24.9	29 (23.4)
Histology	
Adenocarcinoma	84 (67.7)
Squamous carcinoma	37 (29.8)
Other histology ^a)^	3 (2.4)
TNM stage	
III	30 (24.2)
IV	94 (75.8)
ECOG PS	
** 0**	37 (29.8)
** 1**	87 (70.2)
Chemotherapy regimen	
Platinum + pemetrexed	81 (65.3)
Platinum + paclitaxel	19 (15.3)
Platinum + gemcitabine	15 (12.1)
Platinum + docetaxel	9 (7.3)
Response to chemotherapy	
PR	25 (20.2)
SD	75 (60.5)
PD	24 (19.4)
Targeted therapy ^b)^	
No	88 (71.0)
Yes	36 (29.0)
Immunotherapy ^c)^	
No	107 (86.3)
Yes	17 (13.7)
Antiangiogenic therapy ^d)^	
No	89 (71.8)
Yes	35 (28.2)
**PFS (IQR) months**	8.0 (5.0-18.0)
**OS median (IQR) months**	17.0 (12.0-30.0)
At Baseline	
SII (IQR)	809.2 (513.5-1064.2)
PNI (IQR)	47.7 (44.2-52.9)
After 4 cycles	
SII (IQR)	450.2 (287.6-718.9)
PNI (IQR)	43.9 (40.5-52.3)

^a)^ Other histology was pathologically confirmed as non-small cell lung cancer, but the specific was unclear. ^bcd)^ All three regimens were used when progression or relapse has followed a minimum of 4 cycles of first-line platinum-based chemotherapy.

### Relationship between the response of chemotherapy and the SII, PNI

**Figure 1:**
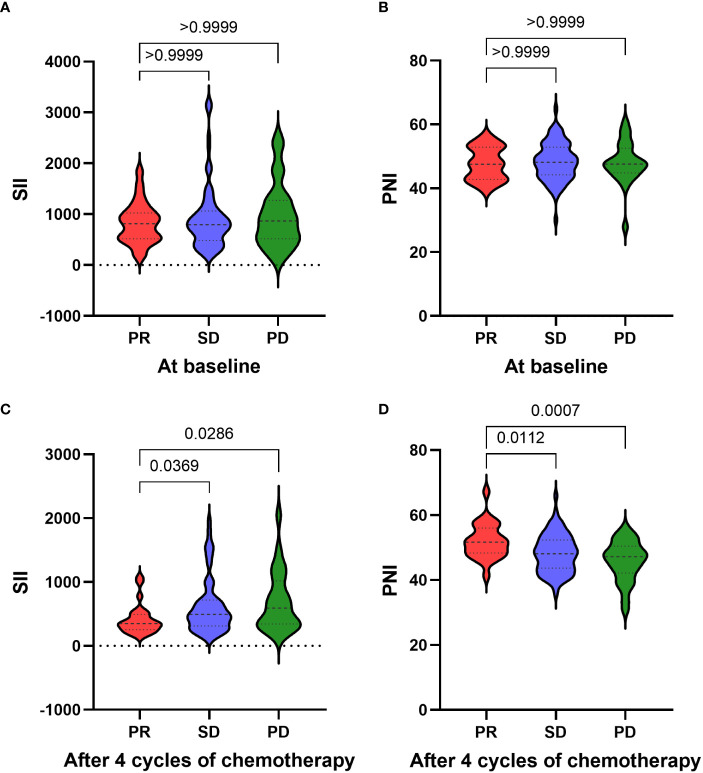
Relationship between the response of chemotherapy and the SII **(A)**/PNI **(B)** at baseline. Relationship between the response of chemotherapy and the SII **(C)**/PNI **(D)** after four cycles of chemotherapy.

According to the tumor response after 4 cycles of platinum-doublet chemotherapy, 124 patients were divided into three groups: PR group (25 cases), SD group (75 cases) and SD group (24 cases). We hypothesized that the patient’s systemic inflammation and immune status may be associated with recent chemotherapy effects, so the relationship between SII/PNI and chemotherapy effect was next assessed. We performed a differential analysis of SII and PNI before and after chemotherapy between the three groups. Before receiving chemotherapy, there was no significant difference in SII between patients in the SD group and PD group compared to the PR group (all p>0.05) (**Figure 1A**). Similarly, there was no significant difference in PNI between three groups (all p>0.05) (**Figure 1B**). After receiving 4 cycles of platinum-doublet chemotherapy, the SII of the SD group (p=0.0369) and PD group (p=0.0286) was significantly different from that of PR group, and the SII of the PR group was significantly lower than that in the SD group and the PD group (**Figure 1C**). At the same time, compared with the PR group, the PNI of the SD group (p=0.0112) and the PD group (p=0.0007) also had a significant difference, and the PNI of the PR group was significantly higher than that of the SD group and the PD group (**Figure 1D**). Therefore, after 4 cycles of chemotherapy, the better the efficacy, the lower the SII, the higher the PNI.

### Relationships between PNI, SII and clinicopathological features after four cycles of chemotherapy

**Figure 2:**
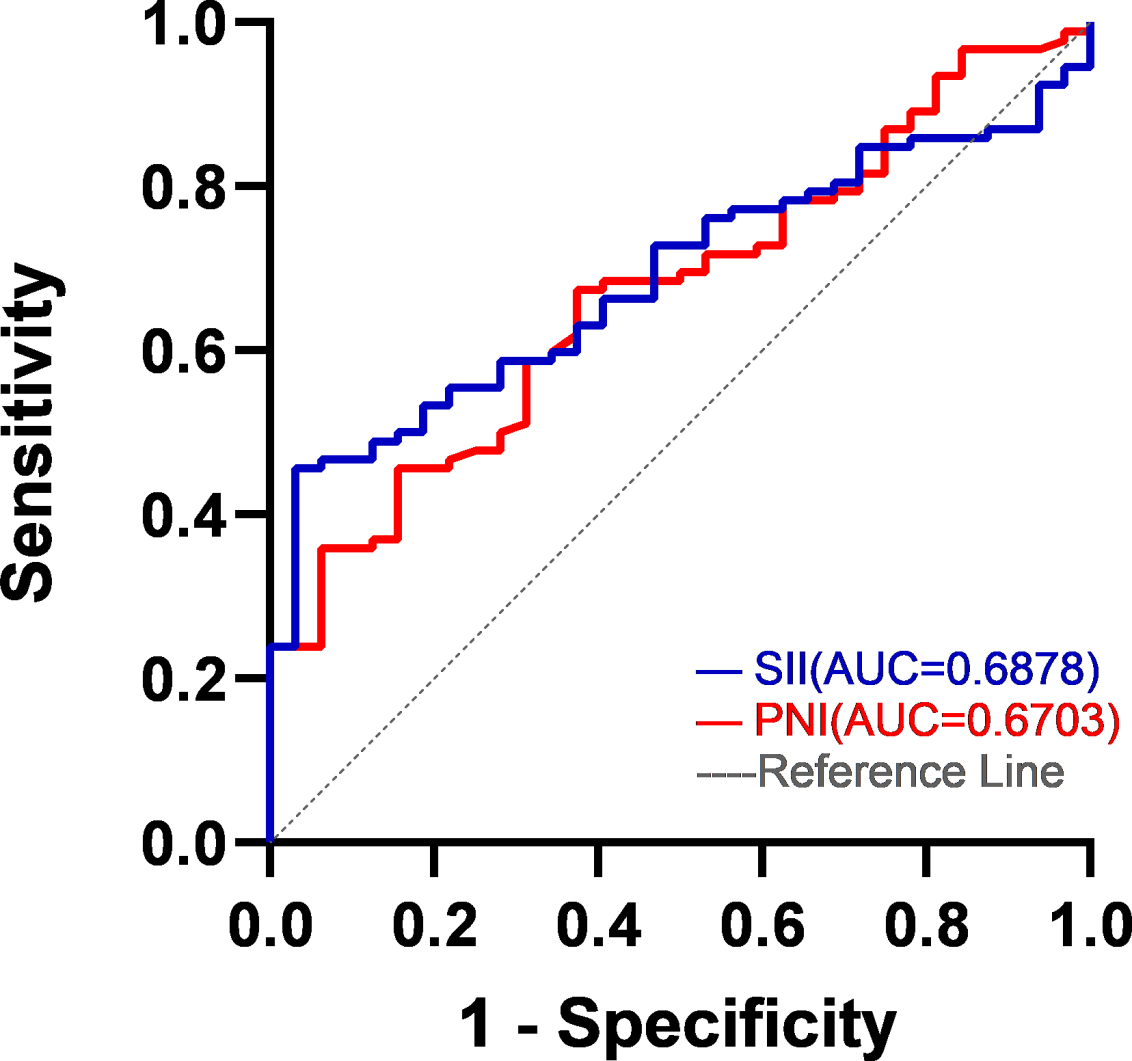
ROC curves of SII and PNI. The AUC of the SII is 0.6878. The AUC of the PNI is 0.6703.

It was taking the SII and PNI after four cycles of chemotherapy as test variables and the survival status of patients during five-year follow-up as state variables, the optimal cut-off values of SII and PNI after four cycles of chemotherapy were determined by the ROC curve to be 593.0 (AUC=0.688; 95% CI, 0.596-0.780; p = 0.002) and 46.4 (AUC=0.670; 95% CI, 0.571-0.770; p = 0.004) (**Figure 2**). According to SII and PNI, the patients were divided into three groups: 0, high SII and low PNI (n=57); 1, high SII or low PNI (n=44); 2, low SII or high PNI (n=23). The correlation analysis between SII-PNI score after four cycles of chemotherapy and clinicopathological features showed no significant difference in gender, age, smoking history, BMI group, pathological type, TNM stage, ECGO PS and chemotherapy regimen among the three groups (all p>0.05). There was no significant correlation between whether or not to receive targeted therapy, immunotherapy, and antiangiogenic therapy in the later stage and the SII-PNI score after 4 cycles of chemotherapy (all p>0.05). However, it was significantly correlated with chemotherapy response after four cycles (χ ^2^ = 14.3, p=0.006) (**Table 2**).

**Table 2:** The clinicopathological characteristics according to SII-PNI scores.

Characteristics	SII-PNI score			χ2	p-Value
	0 (n=57)	1 (n=44)	2 (n=23)		
Gender				1.566	0.457
Male	45	31	19		
Female	12	13	4		
Age (y)				2.936	0.231
<64	29	27	9		
≥64	28	17	14		
Smoking history					
Ever	22	17	14	3.792	0.150
Never	35	27	9		
BMI				1.226	0.874
<18.5	3	4	1		
18.5–24.9	42	29	6		
≥25	12	11	6		
Histology				7.153	0.128
Adenocarcinoma	42	30	12		
Squamous carcinoma	15	13	9		
Other histology	0	1	2		
TNM stage				2.602	0.272
III	16	7	7		
IV	41	37	16		
ECOG PS				1.288	0.525
0	15	13	9		
1	42	31	14		
Chemotherapy regimen				1.526	0.958
Platinum + pemetrexed	38	28	15		
Platinum + paclitaxel	10	6	3		
Platinum + gemcitabine	5	7	3		
Platinum + docetaxel	4	3	2		
Response to chemotherapy				14.300	0.006
PR	19	4	2		
SD	32	27	16		
PD	6	13	5		
Targeted therapy				1.397	0.497
No	39	34	15		
Yes	18	10	8		
Immunotherapy				5.095	0.078
No	45	40	22		
Yes	12	4	1		
Antiangiogenic therapy				3.436	0.179
No	40	29	20		
Yes	17	15	3		

### Survival outcomes of patients in different SII-PNI scores

**Figure 3:**
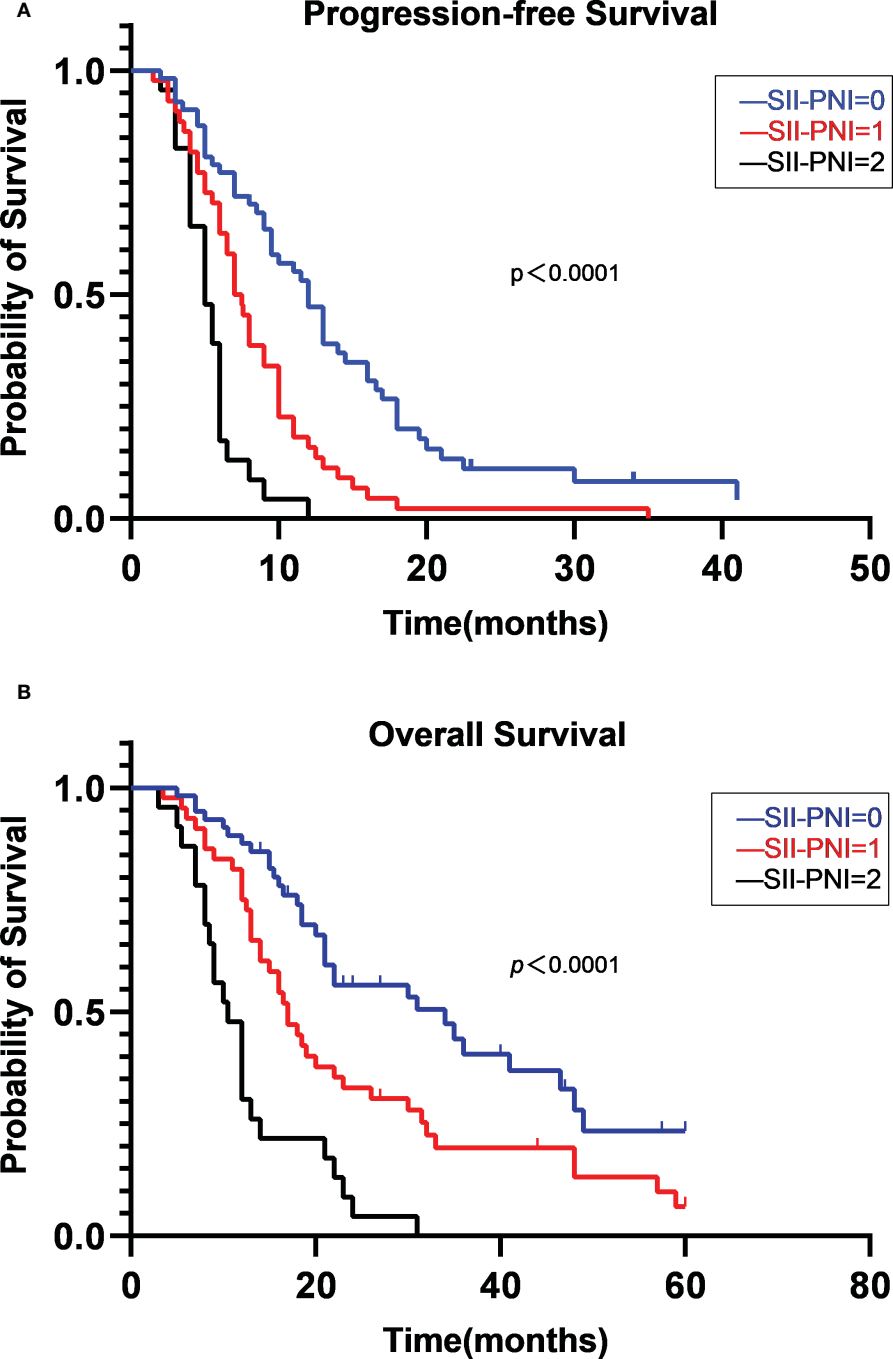
Kaplan-Meier survival analyses of PFS **(A)** and OS **(B)** stratified by SII-PNI score.

All patients were followed up. The median PFS was 8.0 months (95% CI, 6.656-9.344). The PFS of patients with SII-PNI scores of 0, 1, 2 were 12.0, 7.0, 5.0 months, respectively, and there were significant differences among the three groups (all p< 0.001) (**Figure 3A**). The 1-year PD rate was 72.9% of the patients in the whole group. The median OS was 19.0 months (95% CI, 15.779-22.221). The 1-year, 3-year, and 5-year survival rates of all patients were 72.4%, 24.5%, and 11.4%, respectively. Regarding the 5-year survival rate, the SII-PNI score of 0 is 23.4%, while the SII-PNI scores of 1 and 2 was 6.6% and 0. The OS of patients with SII-PNI scores of 0, 1, 2 were 34.0, 17.0, 10.5 months, respectively, and there were significant differences among the three groups (all p < 0.001) (**Figure 3B**).

We performed Cox regression analyses for PFS and OS. **Table 3** summarizes the univariate and multivariate analyses for PFS. Multivariate analysis showed that chemotherapy response of PD (HR, 5.592; 95% CI, 2.770-11.288; p<0.001), SII-PNI score of 1 (HR, 1.814; 95% CI, 1.142-2.881; p=0.012), and SII-PNI score of 2 (HR, 5.344; 95% CI, 2.910-9.814; p<0.001) were independently associated with a shorter PFS. The use of targeted drugs, immune checkpoint inhibitors, and antiangiogenic drugs were protective factors for PFS, but there was no significant statistical significance (all p>0.05). **Table 4** summarizes univariate and multivariate analyses for OS. After multivariate analyses, chemotherapy response of PD (HR, 3.508; 95% CI, 1.546-7.960; p=0.003) and SII-PNI score of 2 (HR, 4.732; 95% CI, 2.561-8.743; p < 0.001) were independently associated with a shorter OS. We also found that the uses of targeted drugs (HR, 0.543; 95% CI, 0.329-0.898; p=0.017) and immune checkpoint inhibitors (HR, 0.218; 95% CI, 0.081-0.584; p=0.002) were protective factors for OS in patients with NSCLC.

**Table 3:** Factors associated with progression-free survival.

Variable	Univariate analysis		Multivariate analysis	
	HR (95% CI)	p-value	HR (95% CI)	p-value
Gender				
Male	Reference			
Female	0.879 (0.526-1.471)	0.625		
Age (y)				
<64	Reference			
≥64	1.044 (0.696-1.567)	0.835		
Smoking history				
Never	Reference			
Ever	1.391 (0.885-2.188)	0.153		
BMI				
<18.5	Reference			
18.5–24.9	1.185 (0.488-2.876)	0.707		
≥25	0.993 (0.386-2.556)	0.988		
Histology				
Adenocarcinoma	Reference			
Squamous carcinoma	1.222 (0.765-1.951)	0.402		
Other histology	2.809 (0.778-10.150)	0.115		
TNM stage				
III	Reference			
IV	1.118 (0.684-1.826)	0.656		
ECOG PS				
0	Reference			
1	0.805 (0.535-1.209)	0.296		
Chemotherapy regimen				
Platinum + pemetrexed	Reference		Reference	
Platinum + paclitaxel	1.203 (0.699-2.070)	0.504	1.184 (0.661-2.119)	0.570
Platinum + gemcitabine	1.138 (0.641-2.020)	0.658	1.169 (0.629-2.173)	0.622
Platinum + docetaxel	1.182 (0.588-2.376)	0.639	1.379 (0.656-2.899)	0.396
Response to chemotherapy				
PR	Reference		Reference	
SD	1.166 (0.660-2.059)	0.597	1.192 (0.689-2.065)	0.530
PD	5.555 (2.758-11.187)	<0.001	5.592 (2.770-11.288)	<0.001
Targeted therapy				
No	Reference		Reference	
Yes	0.791 (0.529-1.185)	0.256	0.740 (0.476-1.150)	0.180
Immunotherapy				
No	Reference		Reference	
Yes	0.870 (0.511-1.480)	0.607	0.670 (0.359-1.252)	0.210
Antiangiogenic therapy				
No	Reference		Reference	
Yes	1.066 (0.713-1.594)	0.755	1.333 (0.843-2.110)	0.219
SII-PNI score				
0	Reference		Reference	
1	1.922 (1.210-3.051)	0.006	1.814 (1.142-2.881)	0.012
2	4.855 (2.656-8.874)	<0.001	5.344 (2.910-9.814)	<0.001

**Table 4:** Factors associated with overall survival.

Variable	Univariate analysis		Multivariate analysis	
	HR (95% CI)	p-value	HR (95% CI)	p-value
Gender				
Male	Reference			
Female	1.350 (0.827-2.205)	0.230		
Age (y)				
<64	Reference			
≥64	1.022 (0.657-1.591)	0.923		
Smoking history				
Never	Reference			
Ever	0.723 (0.436-1.199)	0.209		
BMI				
<18.5	Reference			
18.5–24.9	0.643 (0.262-1.579)	0.336		
≥25	0.540 (0.206 -1.415)	0.210		
Histology				
Adenocarcinoma	Reference			
Squamous carcinoma	1.110 (0.663-1.858)	0.693		
Other histology	2.984 (0.853-10.438)	0.087		
TNM stage				
III	Reference			
IV	1.632 (0.935-2.850)	0.085		
ECOG PS				
0	Reference			
1	0.765 (0.489-1.195)	0.239		
Chemotherapy regimen				
Platinum + pemetrexed	Reference		Reference	
Platinum + paclitaxel	0.880 (0.449-1.726)	0.710	0.869 (0.417-1.811)	0.708
Platinum + gemcitabine	0.940 (0.506-1.747)	0.844	0.876 (0.475-1.860)	0.707
Platinum + docetaxel	1.421 (0.677-2.981)	0.353	1.384 (0.623-3.075)	0.425
Response to chemotherapy				
PR	Reference		Reference	
SD	0.865 (0.451-1.660)	0.663	1.348 (0.704-2.581)	0.368
PD	2.149 (1.006-4.591)	0.048	3.508 (1.546-7.960)	0.003
Targeted therapy				
No	Reference		Reference	
Yes	0.583 (0.364-0.932)	0.024	0.543 (0.329-0.898)	0.017
Immunotherapy				
No	Reference		Reference	
Yes	0.240 (0.097-0.593)	0.002	0.218 (0.081-0.584)	0.002
Antiangiogenic therapy				
No	Reference		Reference	
Yes	0.667 (0.416-1.072)	0.094	0.991 (0.585-1.677)	0.972
SII-PNI score				
0	Reference		Reference	
1	1.729 (1.031-2.899)	0.038	1.409 (0.828-2.399)	0.207
2	6.011 (3.183-11.354)	<0.001	4.732 (2.561-8.743)	<0.001

## Discussion

Our present study showed that higher SII-PNI score are significantly associated with shorter PFS and OS in patients with advance NSCLC and are independent influencing factors of overall survival. To our knowledge, this is the first study to demonstrate that a high SII-PNI score after 4 cycles of platinum-doublet chemotherapy is a reliable prognostic indicator of advanced NSCLC.

Immune status, including inflammatory status and nutritional status, is an essential factor in predicting the prognosis of patients with malignant tumors. As a cancer marker, inflammation has been found to play an essential role in tumorigenesis, progression, and anti-tumor therapy. Inflammation can provide bioactive molecules for the tumor microenvironment, and the products of the inflammatory process are considered potential biomarkers (25, 26). Several studies have explored the relationship between inflammatory indexes and the prognosis of patients with malignant tumors. Hirahara T. et al. have shown that the combination of NLR and PLR can predict chemotherapy response and prognosis in patients with advanced gastric cancer (27). Yao et al. found that in patients with advanced NSCLC who received platinum-doublet chemotherapy, high NLR was significantly correlated with chemotherapy resistance and worse PFS and OS (28). Although NLR and PLR can help evaluate the prognosis of tumor patients, these two indicators integrate only two cell types. Compared with NLR and PLR, SII is composed of peripheral blood neutrophils, lymphocytes, and platelets, which can be used to evaluate systemic inflammatory response. High SII is an independent adverse prognostic indicator for cancer patients. De Giorgi U et al. found that high SII is one of the key prognostic factors of OS in patients with renal cell carcinoma treated with nivolumab (29). In addition, the nutritional status of patients is also an important factor affecting tumor progression. Pre-treatment BMI and albumin levels will affect the prognosis of patients with lung cancer (30–32). As a new type of nutrition monitoring index, PNI is composed of albumin and lymphocyte count. Many studies have proved higher PNI tends to be associated with better prognosis in different tumors (33, 34).

In recent years, there has been an increase in research on the application of SII and PNI in lung cancer. Tong et al. observed that high SII before treatment was associated with radiotherapy and chemotherapy resistance and worse OS in patients with locally advanced NSCLC (35). Liu et al. confirmed that patients with metastatic non-small cell lung cancer had longer PFS and OS in the low-SII group treated with nivolumab (36). Wang et al. have shown that higher PNI before treatment is associated with longer OS in NSCLC patients receiving platinum chemotherapy (37). Xu et al. proved that low PNI is an independent adverse prognostic factor in patients with NSCLC bone metastasis (17). Differing from their findings, our study showed no significant correlation between SII, PNI and PFS, OS at baseline. Khunger M et al. found patients with advanced NSCLC treated with nivolumab, patients in the higher NLR group after 2 cycles of treatment had a poorer prognosis, while pre-treatment NLR was not associated with prognosis (38). Putzu C et al. found none of the indexes evaluated at baseline showed any association with the prognosis in patients receiving anti-PD-1 inhibitor therapy, while the NLR, PLR at 6 weeks were significantly associated with both PFS and OS (39). The study by Suh KJ et al. had similar results (40). Their findings suggest that post-treatment inflammatory markers can predict prognosis for patients with tumors. Therefore, we investigated the relationship between SII, PNI and survival outcomes in patients with advanced NSCLC after 4 cycles of platinum-doublet chemotherapy. The results showed that the SII, PNI after 4 cycles of chemotherapy were correlated with PFS and OS. These inconsistent results remain to be clarified, and we speculate that differences in patient race, pathology type, treatment regimen, sample size, and optimal cut-off values may be one of the reasons. This difference also suggests that changes in inflammatory markers may be more effective in predicting patient outcomes over the course of receiving anti-tumor therapy. Maccio A et al. confirmed the presence of cancer cells, activation of the immune system, and release of inflammatory factors together determine specific changes in energy metabolism, resulting in cachexia in tumor patients and promoting tumor growth; and effective antineoplastic therapies can reverse cachexia status in cancer patients (41). As our findings show, after receiving 4 cycles of chemotherapy, patients had a significant decrease in SII levels and a significant increase in PNI levels (p<0.05). Therefore, we believe that effective chemotherapy can reverse inflammation and nutritional status and counteract the major catabolic drivers that influence nutritional status (i.e., tumor cell and tumor-associated inflammation). The reversibility of effective chemotherapy to inflammation and nutritional status was more significant than baseline in predicting outcomes.

As mentioned earlier, studies have shown that the use of SII and PNI alone can predict the prognosis of lung cancer. Studies have shown that combining SII with PNI to form a SII-PNI score can more fully reflect a patient’s immune status. We found that to date, only four studies have reported on SII-PNI score predicting treatment outcomes in patients with gastric cancer and urothelial carcinoma (21–24). They found that pre-treatment SII-PNI score was an important indicator of chemotherapy sensitivity in gastric cancer and urothelial carcinoma patients treated with combination therapy. There are currently no studies using SII-PNI score to predict lung cancer prognosis. We conducted this study for the first time in patients with advanced NSCLC. Our findings showed no statistically significant differences in SII-PNI scores after 4 cycles of platinum-doublet chemotherapy between gender, age, smoking history, BMI group, pathology type, TNM stage, ECOG PS and chemotherapy regimen, but were significantly correlated with chemotherapy response after 4 cycles. Prognostic analysis showed a worse prognosis for patients with a high SII-PNI score after 4 cycles of platinum-doublet chemotherapy. Chemotherapy response of PD, SII-PNI score of 2 were independent risk factors for PFS and OS; the uses of targeted drugs and immune checkpoint inhibitors were independent protective factors for OS.

We believe that the SII-PNI score is an effective indicator of prognosis in patients with NSCLC. Because the SII-PNI score also reflects the patient’s inflammatory response, immune level and nutritional status. A higher SII-PNI score means that the neutrophil or platelet count in the peripheral blood is higher relative to the lymphocyte count. Neutrophilia leads to increased production of related inflammatory factors, such as pro-angiogenic factors, growth factors, etc. They promote tumors by inhibiting apoptosis, promoting tumor angiogenesis, and stimulating metastasis (42). Neutrophils can also inhibit the cytotoxic effect mediated by lymphokine-activated killer cells, thus down-regulating patients’ anti-tumor cellular immune response (39). The increased platelet count promotes tumor development by promoting tumor cell diffusion, regulating tumor angiogenesis, and promoting immunosuppression (43). Lymphocytes, as critical immune cells, play an important role in immune monitoring. A relative decrease in lymphocytes suggests that the patient may have immunosuppression or deficiency, which promotes tumor progression and bad prognosis. The decrease in serum albumin reflects malnutrition and low immunity, which will increase the complications of the tumor and reduce the tolerance to treatment.

Proteomic and genetic analysis, which is currently in use, cannot be widely used due to its high technical requirements and high cost. The SII-PNI score is a simple, cheap, and easy-to-generalizable indicator that can dynamically reflect the patient’s immune status and treatment effect, which has high guiding value for the selection and adjustment of treatment options, and can effectively predict the long-term prognosis of patients. Based on the current findings, we will further explore the application value of SII-PNI score in NSCLC patients receiving other treatment regimens in the future.

There are also some limitations in our present study. Our study is a retrospective analysis of a single-center small sample and lacks an externally validated model. Therefore, prospective large randomized controlled trials are needed to further confirm the predictive value of the SII-PNI score on the prognosis of patients with NSCLC, solve the problem of inconsistent optimal cut-off values of SII, PNI. In addition, our study only included patients with NSCLC who were treated with platinum-doublet chemotherapy as first-line treatment, and in the future we need to verify whether SII-PNI scores can also predict survival outcomes in NSCLC receiving targeted therapy and immune checkpoint therapy.

## Conclusion

Clinical treatment decisions of patients with advanced NSCLC depend on appropriate prognostic indicators, which can guide clinicians in predicting the efficacy of antineoplastic therapy. SII-PNI score is economical and convenient. It can reflect the immediate immune status of tumor patients in diagnosis and therapy. There is no study on the correlation between SII-PNI score and the prognosis of advanced NSCLC patients receiving chemotherapy alone. We confirmed this association for the first time in advanced NSCLC. Our study found that SII-PNI scores of NSCLC patients after four cycles of platinum-doublet chemotherapy can effectively predict the PFS and OS of patients. This is of great significance for the stratification and final individualized treatment of NSCLC patients.

## Data availability statement

The raw data supporting the conclusions of this article will be made available by the authors.

## Ethics statement

The studies involving human participants were reviewed and approved by The Medical Ethics Committee of Suzhou Municipal Hospital (K-2021-084-H01). Written informed consent for participation was not required for this study in accordance with the national legislation and the institutional requirements.

## Author contributions

RF: collected the date, followed up, made statistics and charts, drafted the manuscript. YC: provided the methodology, participated in research design and manuscript revision. GX and YL: participated in the statistical analyses, revised the manuscript. WP: participated in data collection and data interpretation. ZZ: revised the draft, supervised the work. All authors contributed to the article and approved the submitted version.
